# AMP‐activated protein kinase regulates lipid metabolism and the fibrotic phenotype of hepatic stellate cells through inhibition of autophagy

**DOI:** 10.1002/2211-5463.12221

**Published:** 2017-05-11

**Authors:** Ming Chen, Jiaxing Liu, Lili Yang, Wenhua Ling

**Affiliations:** ^1^ Department of NutritionSchool of Public HealthSun Yat‐Sen UniversityGuangzhouGuangdongChina; ^2^ Guangdong Provincial Key Laboratory of Food, Nutrition and HealthGuangzhouGuangdongChina

**Keywords:** AMP‐activated protein kinase, autophagy, hepatic stellate cell, microtubule‐associated protein light chain 3

## Abstract

Hepatic stellate cells (HSCs) are the principal hepatic cell type responsible for liver fibrosis. Although AMP‐activated protein kinase (AMPK) is known to regulate the activation of HSCs, little is known about its underlying molecular mechanisms. In the present study, we demonstrate that AMPK activation by 5‐aminoimidazole‐4‐carboxamide‐1‐4‐ribofuranoside (AICAR) restricts the fibrotic potential elicited by transforming growth factor β (TGF‐β) in LX‐2 cells through modulation of autophagy. AICAR treatment activated the mechanistic target of rapamycin/Akt pathway and thus inhibited autophagy flux and lipid droplet degradation in lysosomes induced by TGF‐β. Pretreatment with the autophagy inducer rapamycin reversed the effects of AMPK, further confirming that AICAR inhibited TGF‐β‐induced HSC activation via the regulation of autophagy flux. Our study indicates that AICAR exerts its anti‐fibrotic and anti‐lipid depletion effect, at least in part, by inhibiting TGF‐β‐induced autophagy flux.

**Abbreviations**
AICAR: 5‐aminoimidazole‐4‐carboxamide‐1‐4‐ribofuranoside
AMPK: AMP‐activated protein kinase
CQ: chloroquine
GAPDH: glyceraldehyde 3‐phosphate dehydrogenase
HRP: horseradish peroxidase
HSC: hepatic stellate cell
LD: lipid droplet
LY: LY294002
mTOR: mechanistic target of rapamycin
TGF‐β: transforming growth factor β
α‐SMA: α‐smooth muscle actin

Liver fibrosis represents an adaptive response to repeated chronic liver injuries, primarily caused by chronic viral hepatitis and steatohepatitis associated with either alcohol or obesity 1, 2. Formation and activation of collagen‐producing myofibroblasts play critical roles in the pathogenesis of liver fibrosis. Myofibroblasts originate from distinct cellular populations, including liver‐resident hepatic stellate cells (HSCs), portal fibroblasts and bone‐marrow‐derived collagen‐producing cells. Activated HSCs are the major source of myofibroblasts in the development of liver fibrosis 3. Upon chronic liver injury, HSCs, a non‐parenchymal cell located perisinusoidally in the space of Disse between hepatic endothelial cells and hepatocytes, transform from a quiescent phenotype into an activated phenotype characterized by excess proliferation, loss of lipid droplets (LDs) and consequent retinoid reserve, and overexpression of extracellular matrix proteins, such as α‐smooth muscle actin (α‐SMA) and collagen type I. Thus, HSCs represent a major anti‐fibrotic target for the treatment of hepatic fibrogenesis.

Autophagy is a general process by which cytoplasmic cargos, including damaged organelles and misfolded or long‐lived proteins, reach lysosomes for degradation 4. To date, three types of autophagy have been identified, namely macroautophagy, microautophagy and chaperone‐mediated autophagy. They differ from each other with respect to delivery method of cargo to lysosomes. Herein, we focus on macroautophagy (hereafter referred to as autophagy). Autophagy is an important player in various diseases such as infections, cancer, aging and neurodegeneration 5, 6. In liver, autophagy is involved in alcoholic fatty liver disease, drug‐induced liver injury, ischemia–reperfusion injury, hepatic cirrhosis and carcinoma 7, and therefore represents a promising therapeutic target for the treatment of some liver diseases. Autophagy has been reported to promote HSC survival and activation during *in vitro* activation 8. Inhibition of autophagy by the pharmacological reagent chloroquine (CQ) helps to restore lipid content in HSCs 9. Interestingly, recent studies found that autophagy could, alternatively, stimulate autophagic cell death in LX‐2 cells or rat primary HSCs treated by sorafenib or nilotinib 10, 11. Therefore, the contribution of autophagy to HSC activation appears to depend on cell context and type of stimulus.

AMP‐activated protein kinase (AMPK) is a highly conserved protein kinase in mammalian cells, comprising a heterotrimeric complex of a catalytic α‐subunit and regulatory β‐ and γ‐subunits. AMPK can be activated by direct phosphorylation by the upstream kinase liver kinase B1 at the site of Thr172 on the α‐subunit, or through indirect allosteric changes of the γ‐subunit, thereby regulating the phosphorylation state of the α‐subunit. These conformational changes involve an increase in the AMP/ATP ratio intracellularly and therefore are the basis of many AMPK activators, such as 5‐aminoimidazole‐4‐carboxamide‐1‐4‐ribofuranoside (AICAR) and metformin. Generally speaking, AMPK activation switches off almost all anabolic pathways and promotes mitochondrial biogenesis and corresponding nuclear receptors, enzymes and binding proteins involved in fatty acid β‐oxidation in mitochondria to maintain the cellular ATP level 12, 13, thereby lowering lipid content in the organism. However, in some other situations, AMPK restores the lipid content 14. It is possible that the contribution of AMPK activation to lipid metabolism is cell and tissue specific.

Previous studies have shown that activation of AMPK by AICAR or metformin inhibits platelet‐derived growth factor‐induced proliferation of primary human HSCs 15 or the human HSC cell line hTERT 16. A recent study further demonstrated that AMPK activation also exerts an anti‐fibrotic effect in LX‐2 cells under transforming growth factor β (TGF‐β)‐stimulated conditions by physical interaction with p300 17. Here, we explored the role of AICAR on autophagy in the regulation of LX‐2 cell activation.

## Materials and methods

### Reagents

AMP‐activated protein kinase (cat. no. 2532, 1 : 1000) and Thr‐172 AMPK antibodies (cat. no. 2535, 1 : 1000) were obtained from Cell Signaling Technology (Danvers, MA, USA). All horseradish peroxidase (HRP)‐linked secondary antibodies were purchased from Santa Cruz Biotechnology (Dallas, TX, USA). AICAR (cat. no. A9978) and α‐SMA (cat. no. A2547, 1 : 5000) were from Sigma‐Aldrich (St Louis, MO, USA). Lipofectamine 2000 (cat. no. 11668019), Bodipy 493/503 (cat. no. D3922), Lysotracker red (cat. no. L7528), ProLong Gold Antifade Reagent (cat. no. P36931) and Alexa Flour 633 coupled anti‐mouse secondary antibody (cat. no. A‐21052) were purchased from Thermo Fisher Scientific (Waltham, MA, USA). TGF‐β (240‐B) was purchased from R&D Systems (Minneapolis, MN, USA). Unless indicated, other chemicals were obtained from Sigma‐Aldrich. The human LX‐2 cell line was a gift from S. Friedman (Mount Sinai School of Medicine) 18.

### Cell culture and treatment

Human stellate cell line LX‐2 was maintained in Dulbecco's modified Eagle's medium containing 2% FBS and 1% penicillin/streptomycin in a humidified atmosphere containing 5% CO_2_ at 37 °C. For treatment experiments, cells were stimulated with TGF‐β or TGF‐β plus AICAR at the indicated concentrations for 24 h. In some experiments, prior to AICAR treatment, LX‐2 cells were pretreated with CQ for 4 h.

### Immunoblot and immunofluorescence

Total protein concentrations were determined using the BCA Protein Assay Kit (Pierce; Thermo Fisher Scientific). Equal amounts of proteins were separated using SDS/PAGE and then transferred to a poly(vinylidene difluoride) membrane (Millipore, Billerica, MA, USA). Unspecific binding sites were blocked with 5% non‐fat milk in Tris Buffered saline with Tween 20 detergent (TBST) for 1 h, and subsequently incubated with corresponding primary antibodies overnight followed by three washes in TBST for 5 min each. HRP‐conjugated secondary anti‐rabbit, anti‐mouse antibodies were applied for 1 h, followed by three washes in TBST for 5 min each. Blots were then incubated with ECL (Pierce; Thermo Fisher Scientific) for 1 min before exposure to film. The intensity of bands was quantified by imagej software (NIH, Bethesda, MD, USA).

For immunofluorescence, cells were plated on glass coverslips, and then stained with primary antibody to α‐SMA (1 : 400) as previously described 19. For the staining of α‐SMA, cells were fixed with methanol at −20 °C for 5 min. Corresponding Alexa Flour 633‐coupled anti‐mouse antibody was used to visualize the antibody binding. LDs were stained with Bodipy 493/503 (1 μg·mL^−1^) for 30 min. Lysosomes were stained with Lysotracker red. Following mounting with ProLong Gold anti‐fade reagent with DAPI overnight, images were taken using a Leica SP5 confocal microscope (Leica Microsystems, Wetzlar, Germany). The number of LDs was analyzed using imagej software.

### Plasmid preparation and transfection

Plasmids mRFP‐GFP‐LC3B (cat. no. 21074, Addgene, Cambridge, MA, USA) were extracted by using PureLink HiPure plasmid midiprep kit (Invitrogen; Thermo Fisher Scientific), dissolved into Tris‐EDTA buffer and quantified with a BioPhotometer (Eppendorf, Hamburg, Germany). For transfection experiments, LX‐2 cells were cultured on glass coverslips and transfected with 2 μg plasmid using Lipofectamine 2000. After treated with AICAR or TGF‐β, cells were observed under confocal microscopy (Leica SP5) for exogenous tandem fluorescent LC3B. Quantification of autophagosome and autolysosome dots was performed using imagej software.

### Statistics

Data are presented as means ± SEM. Statistical analyses were performed by ANOVA with Fisher's least significant difference test for *post hoc* analysis or Student's *t* test (spss v16.0; SPSS Inc., Chicago, IL, USA). Graphics were processed by graphpad Prism 5.0 (graphpad Software, La Jolla, CA, USA). A *P* value < 0.05 was considered significant.

## Results

### AMPK phosphorylation inhibits the activation of human hepatic stellate cell line LX‐2

**Figure 1:**
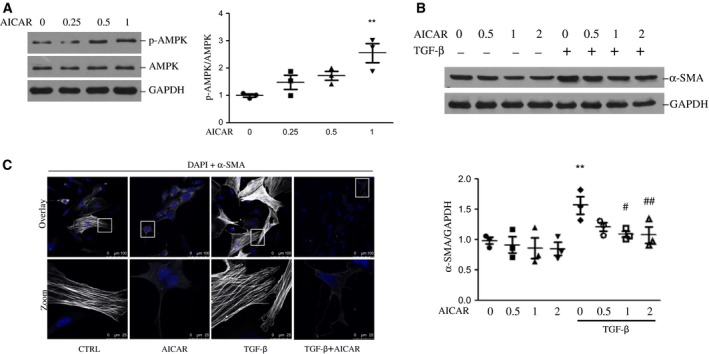
Effect of AICAR treatment on LX‐2 activation in the presence or absence of TGF‐β. (A) Verification of AMPK agonist AICAR on AMPK activation. Representative blots of LX‐2 treated with AICAR for 2 h at the indicated concentrations (0, 0.25, 0.5, 1 mm) are shown. p‐AMPK and AMPK were detected by the indicated antibodies using western blot. Quantification of p‐AMPK/AMPK ratio is shown in the right panel. **P < 0.01 vs control. (B) LX‐2 cells were treated with different concentrations of AICAR (0, 0.5, 1, 2 mm) in the presence or absence of TGF‐β. Western blots were performed with specific indicated antibodies, and data are presented as means ± SEM. Quantitative analyses of α‐SMA expression is shown in the lower panel. **P < 0.05 vs control, ^#^ *P* < 0.05 and ^##^ *P* < 0.01 vs TGF‐β group. GAPDH, glyceraldehyde 3‐phosphate dehydrogenase. (C) Representative fluorescence images of α‐SMA (gray) and DAPI (blue) in LX‐2 cells treated with AICAR (1 mm) in the presence or absence of TGF‐β (4 ng·mL^−1^). Images taken at original magnification (upper panel) and their corresponding magnified images enclosed in white boxes are shown. Scale bar, 100 μm and 25 μm for upper and lower panel, respectively. Experiments were repeated three times.

LX‐2 cells were treated with an increasing dosage of AICAR (0, 0.25, 0.5, 1 mm) with or without 4 ng·mL^−1^ TGF‐β for 24 h. As expected, there was a dose‐dependent increase of phosphorylation of AMPK in response to 0–1 mm AICAR treatment (Fig. 1A). TGF‐β stimulation caused increased fibrotic marker α‐SMA expression up to 1.5‐fold, which was attenuated by co‐incubation of AICAR. Expression of α‐SMA was significantly decreased by AICAR at concentrations of 1 and 2 mm in the presence of TGF‐β (Fig. 1B). To further demonstrate the effect of AMPK on HSC activation, LX‐2 cells were cultured on coverslips and treated with TGF‐β and/or AICAR for 24 h. Immunofluorescence staining showed that LX‐2 displayed a significantly increased expression of α‐SMA protein after TGF‐β treatment, which could be dramatically alleviated by AICAR co‐treatment (Fig. 1C). Taken together, these data implicate a protective effect of AMPK activation in the regulation of LX‐2 cell fibrotic response.

### AICAR treatment inhibited intracellular lipid droplet depletion in LX‐2 cells induced by TGF‐β

**Figure 2:**
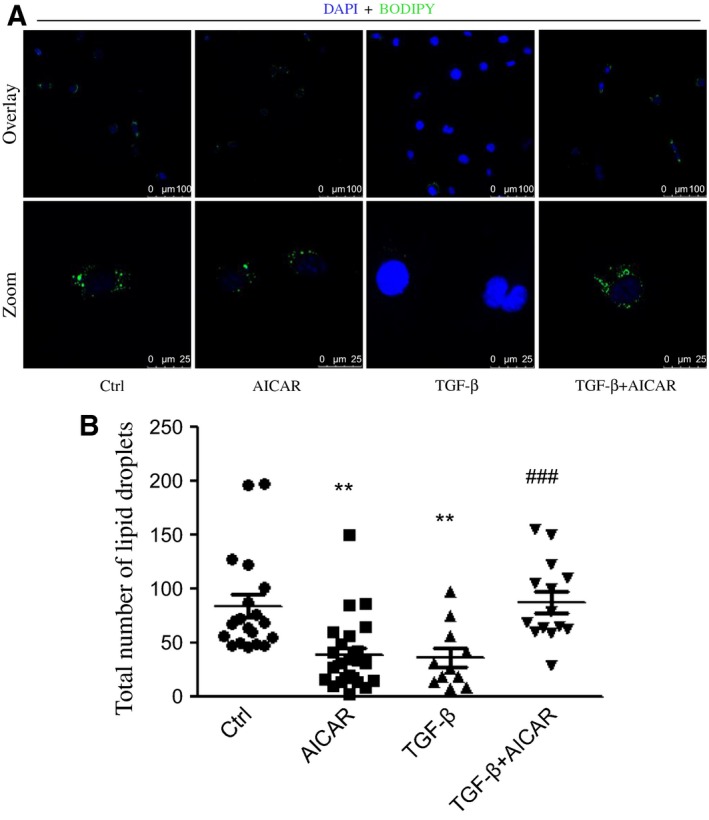
AMPK activation attenuates LD loss induced by TGF‐β in LX‐2 cells. (A) LX‐2 cells were treated with AICAR (1 mm) plus or minus TGF‐β for 24 h. LDs were visualized by staining with Bodipy 493/503 (green), and nuclei were counterstained by DAPI (blue); enlarged areas enclosed in white boxes in upper panel are shown in the lower panel. (B) Quantitative analysis of total number of LDs in (A). **P < 0.01 vs vehicle, ^###^ *P* < 0.001 vs TGF‐β group. Scale bar, 100 μm and 25 μm for upper overlay panel and bottom zoom panel. Experiments were repeated three times.

To investigate the role of AMPK activation in LD metabolism in LX‐2 cells in the presence or absence of TGF‐β, LX‐2 cells were preincubated with retinol and palmitic acid to promote LD formation overnight followed by AICAR treatment or in combination with TGF‐β 20. We observed that TGF‐β significantly reduced the total number of LDs in comparison with vehicle; by contrast, AICAR restored the loss of LDs induced by TGF‐β (Fig. 2A,B).

### AICAR suppressed autophagy in LX‐2 cells through upregulation of the mTOR/Akt pathway

**Figure 3:**
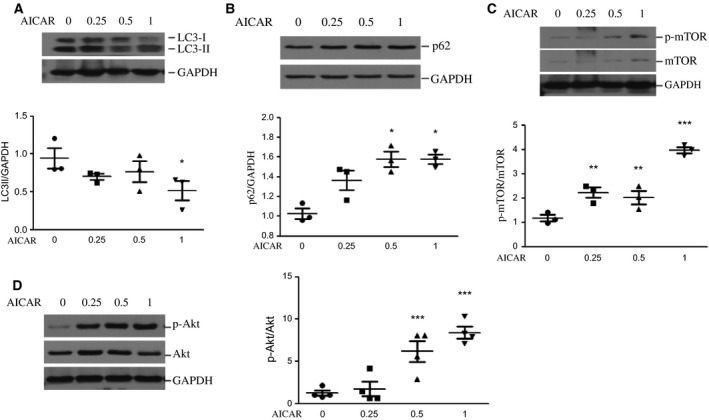
AICAR treatment inhibits autophagy through regulation of the mTOR/Akt signalling pathway. LX‐2 cells were treated with AICAR at the concentration of 0, 0.25, 0.5 and 1 mm for 24 h. Representative blots depicting LC3 (A), p62 (B), total and phosphorylated (Ser2448) mTOR (C) and total and phosphorylated (Thr308) Akt (D) are shown and quantitative analysis is shown in the lower panels (A–C) or right panel (D). *P < 0.05, **P < 0.05, ***P < 0.05 vs vehicle. Experiments were repeated three times.

To explore the role of AMPK activation on autophagy in LX‐2 cells, cells were treated with AICAR at the concentrations of 0, 0.25, 0.5 and 1 mm for 24 h. As shown in Fig. 3A,B, AICAR treatment resulted in a marked decrease in the expression of LC3‐II, a marker of autophagosomes, concomitant with significantly increased expression of p62, a substrate of autophagy, in a dose‐dependent manner. This suggests that AMPK activation in LX‐2 cells inhibits autophagosome formation. To further investigate the mechanism by which AICAR treatment suppressed autophagy, we treated LX‐2 cells as described above and determined the level of mechanistic target of rapamycin (mTOR)/Akt. As demonstrated in Fig. 3C,D, LX‐2 cells showed a significant increase in the expression of both p‐mTOR/mTOR and p‐Akt/Akt, which are negative regulators of autophagosome formation.

### AICAR inhibits TGF‐β‐induced autophagy in LX‐2 cells

**Figure 4:**
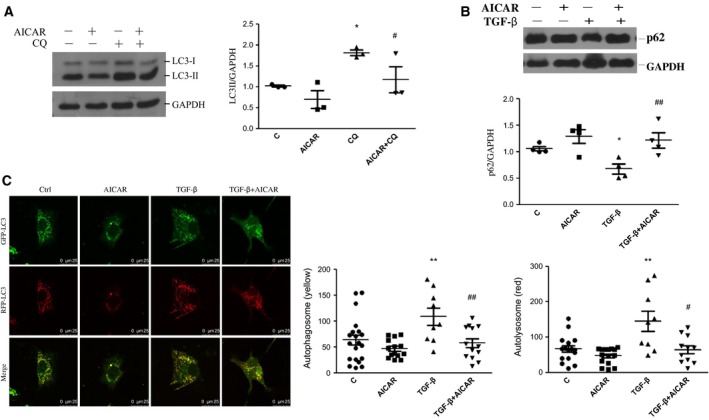
Effect of AICAR treatment on TGF‐β‐induced autophagy activity in LX‐2 cells. (A) LX‐2 cells were incubated with CQ for 4 h and then with AICAR for another 24 h. Quantitative analysis is shown in the right panel. *P < 0.05 vs vehicle, ^#^ *P* < 0.05 vs CQ group. (B) LX‐2 cells were stimulated with AICAR, TGF‐β or their combination for 24 h. Representative blot of p62 is shown and quantitative analysis of p62 expression is shown in the lower panel. *P < 0.05 vs vehicle, ^##^ *P* < 0.01 vs TGF‐β group. (C) mRFP‐GFP‐LC3B plasmid‐transfected cells were stimulated with AICAR, TGF‐β or their combination for 24 h, and then visualized by confocal microscopy with yellow dots and red dots representing autophagosomes and autolysosomes, respectively. Representative images are shown and quantification of autophagosomes and autolysosomes is shown in the right panel. **P < 0.01 vs control, ^#^ *P* < 0.05, ^##^ *P* < 0.01 vs TGF‐β. Scale bar, 25 μm. Experiments were repeated three times.

To further investigate the effect of AICAR on autophagic flux in LX‐2 cells, we pretreated cells with 20 μm of CQ, an autophagy inhibitor, for 4 h, followed by AICAR for another 24 h. As shown in Fig. 4A, expression of LC3B‐II was increased by CQ pretreatment compared with vehicle. When cells were treated with CQ plus AICAR, LC3II expression was dramatically decreased compared with treatment with CQ alone. Next, we examined the effect of AICAR on TGF‐β‐induced autophagy. AICAR inhibited TGF‐β‐induced down‐regulation of p62, which is efficiently degraded by autophagy and inversely correlated with autophagy activity (Fig. 4B). We also confirmed these findings by transfecting LX‐2 cells with mRFP‐GFP‐LC3B (tandem fluorescent LC3B, tfLC3b) plasmid before treatment with AICAR, TGF‐β or their combination. During autophagosome maturation of tfLC3B‐transfected cells, only red signal could be detected as GFP fluorescence was quenched in lysosomes. Therefore, autophagosomes and autolysosomes were labeled with yellow and red dots, respectively. TGF‐β dramatically increased yellow and red dot number, whereas co‐treatment with AICAR significantly attenuated yellow and red puncta number, as demonstrated in Fig. 4C. Collectively, these data demonstrated that AMPK activation by AICAR treatment suppressed TGF‐β‐mediated up‐regulation of autophagy in LX‐2 cells, especially in lysosomal degradation.

### Autophagy is involved in AMPK‐mediated lipid droplets storage and α‐SMA expression in LX‐2 cells

**Figure 5:**
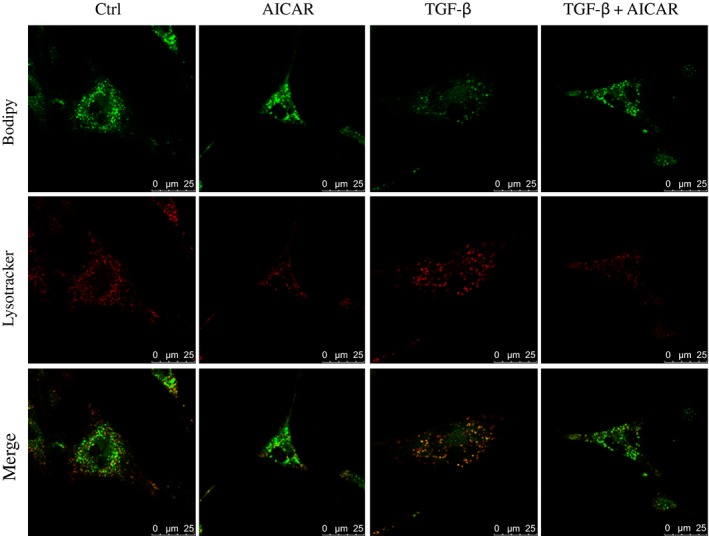
After AICAR, TGF‐β or their combination treatment, LX‐2 cells were stained with Bodipy 493/503 (green) and lysotracker (red), which are dyes of LDs and lysosomes, respectively. Scale bar, 25 μm.

**Figure 6:**
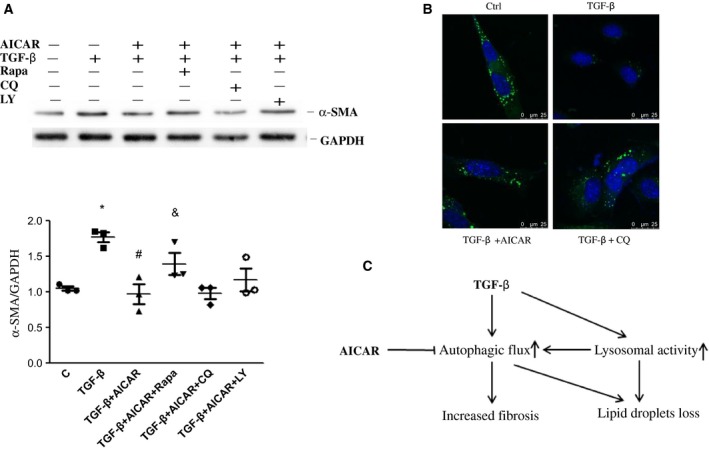
Autophagy is involved in AICAR‐mediated LX‐2 cell activation. (A) After pretreatment with rapamycin (Rapa), CQ and LY294002 (LY) for 4 h, LX‐2 cells were stimulated with AICAR or TGF‐β for another 24 h. α‐SMA protein expression was detected by western blot using specific antibody. Quantitative analysis is shown in the lower panel. *P < 0.05 vs vehicle, ^#^ *P* < 0.05 vs TGF‐β group, ^&^ *P* < 0.05 vs TGF‐β + AICAR. (B) LX‐2 cells were treated with retinol and palmitic acid overnight to promote LD formation before subsequent TGF‐β, AICAR and CQ exposure. After 24 h, cells were fixed by 4% paraformaldehyde and stained with Bodipy 493/503 and DAPI. Scale bar, 25 μm. (C) Simplified model depicting that AICAR inhibited TGF‐β‐mediated up‐regulation of autophagic activity and its role in human HSC line LX‐2 activation. Experiments were repeated three times.

To explore the effect of autophagy on AMPK activation‐mediated LX‐2 cell activation, cells were treated with AICAR, TGF‐β or their combination for 24 h followed by Bodipy and Lysotracker red staining. Co‐localization of LDs and lysosomes was significantly enhanced by TGF‐β stimulation, which was markedly attenuated by co‐incubation of AICAR. These data suggested that AICAR regulated lipid metabolism in LX‐2 cells via an autophagy–lysosome pathway (Fig. 5). Additionally, AICAR treatment abolished TGF‐β‐mediated induction of α‐SMA expression, which could be partly reversed by pretreatment with the autophagy inducer rapamycin. Interestingly, pharmacological inhibition of autophagy by CQ or LY294002 failed to further decrease α‐SMA protein expression in cells elicited by AICAR treatment (Fig. 6A). Taken together, these findings demonstrated that autophagy is involved in AMPK‐mediated HSC activation.

To further investigate whether TGF‐β‐induced LD depletion relies on autophagy, LX‐2 cells were preincubated with the autophagy inhibitor CQ, then stimulated with TGF‐β. As expected, TGF‐β caused LD depletion dramatically, whereas pretreatment of CQ prevented TGF‐β‐induced LD depletion (Fig. 6B). Collectively, these data demonstrated that AMPK activation inhibited TGF‐β‐induced intracellular LD depletion, which relied on increased autophagic flux, and finally inhibited TGF‐β‐induced HSC activation.

## Discussion

Transforming growth factor β is a key cytokine that drives HSC activation following hepatocellular injury. With a human HSC line, LX‐2, we found that TGF‐β treatment led to a further increase in the expression of α‐SMA and contributed to the depletion of LDs, concomitant with an upregulation of autophagy. Co‐incubation with AICAR alleviated TGF‐β‐induced α‐SMA expression and LDs loss through inhibiting autophagy in an mTOR/Akt‐dependent manner. Figure 6C shows a simplified model depicting AICAR inhibition of TGF‐β‐mediated up‐regulation of autophagic activity and its role in human HSC line LX‐2 activation.

Accumulating evidence has shown that AMPK stimulated autophagy 21, 22 through inactivation of mTOR or direct phosphorylation of ULK1, the most upstream component of autophagy in mammalian cells 23, 24. To date, most studies in regard to the interaction of AMPK and autophagy have established that AMPK activation regulates autophagy in multiple tissues and cell types. For example, AMPK activation promoted autophagy, resulting in attenuation of fatty liver of mice by fasting or high fat diet 25, 26. Here, we observed that AMPK inhibited autophagy in HSC line LX‐2 as demonstrated by both biochemical assays and the fluorescence method. It was well established that mTOR/Akt negatively regulated autophagy. We demonstrated that AMPK activation by AICAR in LX‐2 cells suppressed autophagy via upregulation of p‐mTOR/mTOR and p‐Akt/Akt. AMPK is a key energy sensor and modulates cell metabolism to maintain energy homeostasis. During starvation, both AMPK and autophagy are activated to provide energy in order to prevent dysfunction of the organism. It may not be that important for HSCs to stimulate fatty acid β‐oxidation to maintain energy homeostasis. This might explain the results that AMPK activation inhibited rather than activated autophagy in LX‐2 cells. Future studies need to focus on the reasons why AMPK exerts a different effect on autophagy in HSCs vs other cell types. It is also important to figure out whether AMPK activation still inhibits autophagy in HSCs *in vivo*. Additionally, TGF‐β also activates autophagy in a human hepatocellular carcinoma cell line as well as some mammary carcinoma cells, including MDA‐MB‐231 cells 27. Similarly, we found that TGF‐β induced autophagy in LX‐2 cells.

In multiple mammalian tissues, AMPK controls lipid metabolism by integrating nutritional and hormonal signals in peripheral tissues and hypothalamus 28, 29. AMPK suppresses liver lipid production by inhibiting lipogenesis‐associated genes while decreasing lipid deposition via increased lipid oxidation 30, 31. However, AMPK was also thought to inhibit lipolysis in adipocytes since AMPK repressed free fatty acids and glycerol release from 3T3‐L1 cells in the presence of prolipolytic factors such as 3‐isobutyl‐1‐methylxanthine, isoproterenol and forskolin 32. Recently, AMPK was shown to stimulate TG accumulation in passaged rat HSCs, by up‐regulating both mRNA and protein expression of lipogenesis‐related genes, including peroxisome proliferator‐activated receptor γ, sterol regulatory element‐binding protein 1c and CCAAT/enhancer‐binding protein‐α 14. In contrast, we demonstrated that AMPK activation by AICAR prevented lipid depletion in LX‐2 cells in the presence of TGF‐β. We further showed that the autophagy–lysosome pathway participated in this process. Lipophagy is a process in which cytoplasmic LDs are engulfed by an autophagosome and then delivered to lysosomes for degradation. Lipophagy was first described in liver 33. Several reagents, such as caffeine and thyroid hormone, were reported to protect against fatty liver through modulation of lipophagy 34, 35. However, lipophagy in HSCs caused increased free fatty acids by lipolysis, thereby promoting fibrosis 9. Here, we demonstrated that AMPK activation by AICAR restored LDs and inhibited lipophagy, which may partly contribute to a reduced level of fibrosis.

Autophagy has been implicated in liver fibrosis as mice with defective autophagy in HSCs within liver displayed attenuated liver fibrosis following liver injuries. However, *atg5* ^−/−^ mice exposed to chronic carbon tetrachloride administration showed higher levels of fibrogenic markers, suggesting a complicated interaction between liver‐resident cells in response to an autophagy activity change 9, 36. In the present study, we showed that AICAR treatment suppressed TGF‐β‐induced up‐regulation of autophagy, further leading to inhibition of α‐SMA expression elicited by TGF‐β. Pretreatment with the pharmacological activator of autophagy rapamycin partially reversed AICAR treatment‐induced down‐regulation of α‐SMA protein expression.

In summary, our findings provide evidence that autophagy plays an essential role in mediating the anti‐fibrotic and anti‐lipid depletion effect of AMPK in HSCs. Future studies are needed to characterize the relationship between lipid metabolism and fibrogenesis and their roles in mediating the anti‐fibrotic effect of autophagy.

## Author contributions

MC and JXL planned and performed experiments, analyzed data and wrote the paper. LLY and WHL designed the study and wrote the paper.
